# Supplementary material for: Placental malaria induces a unique methylation profile associated with fetal growth restriction
Source: Epigenetics. 2025 Mar 6;20(1):2475276. doi: 10.1080/15592294.2025.2475276 (PMC11901535; doi:10.1080/15592294.2025.2475276)

**Supplemental Table 1. Patient characteristics.**							
**Study ID**	**Study group**	**Collection site**	**Maternal comorbidities**	**Gestational week**	**Infant sex**	**Birth weight (gram)**	**Intergrwoth-21 birth weight percentile**
1	C^{FGR}	United States	anemia, generalized anxiety	39	Female	3505	79.53
2	C^{FGR}	United States	none	39	Male	2895	13.66
3	C^{FGR}	United States	none	39	Male	3625	80.66
4	C^{FGR}	United States	none	39	Male	3815	90.09
5	$C^{PM\text{-}FGR}$	Uganda	none	39	Female	2900	25.41
6	$C^{PM\text{-}FGR}$	Uganda	none	39	Female	2880	20.61
7	$C^{PM\text{-}FGR}$	Uganda	none	38	Female	3650	90.30
8	$C^{PM\text{-}FGR}$	Uganda	none	38	Male	2930	33.42
9	FGR	United States	cHTN, SIPE, gDM, subclinical hypothyroidism	36	Female	2165	6.36
10	FGR	United States	paroxysmal positional vertigo	38	Female	2280	1.62
11	FGR	United States	none	37	Female	2220	3.21
12	FGR	United States	asthma	37	Female	2380	6.01
13	FGR	United States	gHTN, gestational thrombocytopenia	37	Female	2135	2.59
14	FGR	United States	Crohn's disease, opioid use, PreE	38	Male	2425	3.98
15	FGR	United States	gDM, PreE, anemia	36	Male	1430	0.14
16	FGR	United States	gHTN, subclinical hypothyroidism, generalized anxiety, depression	37	Male	1960	0.98
17	PM-FGR	Uganda	anemia	42	Female	2900	6.25
18	PM-FGR	Uganda	none	39	Female	2540	3.14
19	PM-FGR	Uganda	none	39	Female	2500	4.15
20	PM-FGR	Uganda	none	37	Female	2030	1.85
21	PM-FGR	Uganda	anemia	41	Male	3000	7.00
22	PM-FGR	Uganda	anemia	39	Male	2550	3.71
23	PM-FGR	Uganda	none	40	Male	2670	3.27
24	PM-FGR	Uganda	anemia	39	Male	2650	4.01

Supplemental Figure 1.

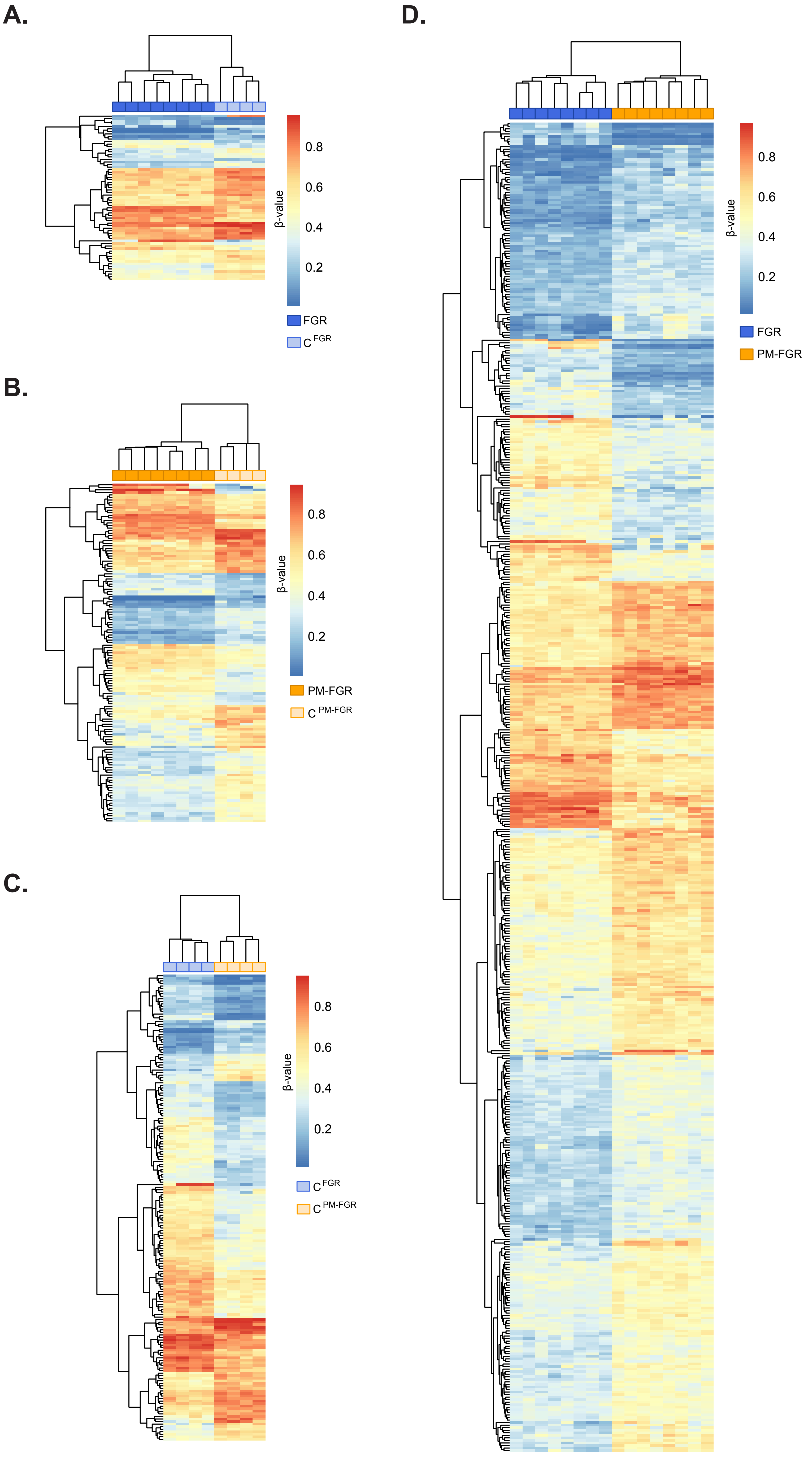

Supplemental Figure 2.

A.

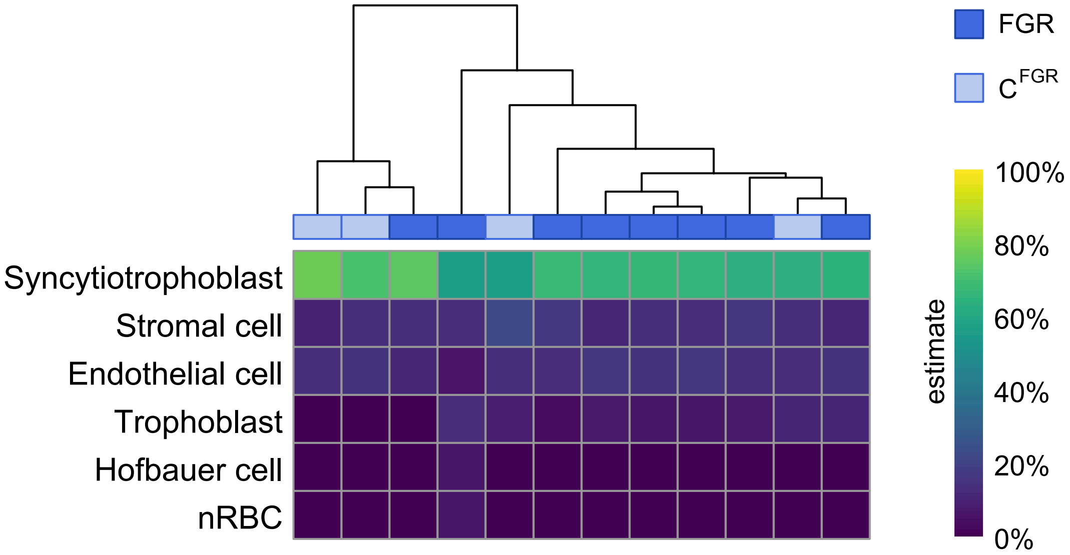

B.

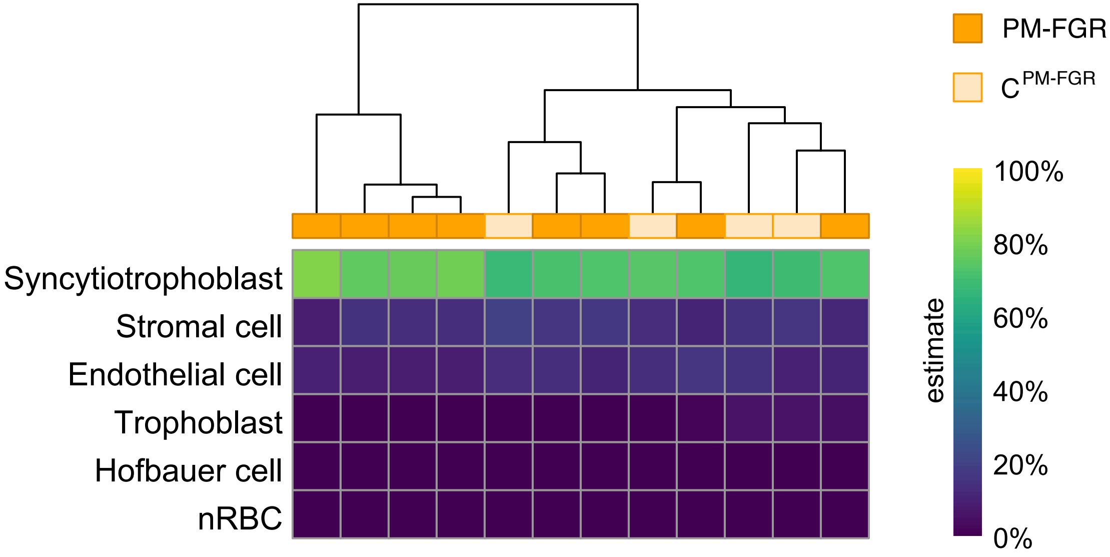

Supplement: Supp Files.pdf [file KEPI_A_2475276_SM1247.pdf]
