# Supplementary material for: Placental malaria induces a unique methylation profile associated with fetal growth restriction
Source: Epigenetics. 2025 Mar 6;20(1):2475276. doi: 10.1080/15592294.2025.2475276 (PMC11901535; doi:10.1080/15592294.2025.2475276)
Supplement: Supplemental Table 1_patient details_1st revision.docx [file KEPI_A_2475276_SM1246.docx]

| **Supplemental Table 1. Patient characteristics.** | | | | | | | | |
| --- | --- | --- | --- | --- | --- | --- | --- | --- |
| **Study ID** | **Study group** | **Collection site** | **Maternal comorbidities** | **Maternal age** | **Gestational week** | **Birth weight (gram)** | **Intergrwoth-21**  **birth weight percentile** | **Infant sex** |
| 1 | C^FGR^ | United States | anemia, generalized anxiety | 29 | 39 | 3505 | 79.53 | Female |
| 2 | C^FGR^ | United States | none | 42 | 39 | 2895 | 13.66 | Male |
| 3 | C^FGR^ | United States | none | 33 | 39 | 3625 | 80.66 | Male |
| 4 | C^FGR^ | United States | none | 40 | 39 | 3815 | 90.09 | Male |
| 5 | C^PM-FGR^ | Uganda | none | 22 | 39 | 2900 | 25.41 | Female |
| 6 | C^PM-FGR^ | Uganda | none | 17 | 39 | 2880 | 20.61 | Female |
| 7 | C^PM-FGR^ | Uganda | none | 23 | 38 | 3650 | 90.30 | Female |
| 8 | C^PM-FGR^ | Uganda | none | 16 | 38 | 2930 | 33.42 | Male |
| 9 | FGR | United States | cHTN, SIPE, gDM, subclinical hypothyroidism | 38 | 36 | 2165 | 6.36 | Female |
| 10 | FGR | United States | paroxysmal positional vertigo | 36 | 38 | 2280 | 1.62 | Female |
| 11 | FGR | United States | none | 27 | 37 | 2220 | 3.21 | Female |
| 12 | FGR | United States | asthma | 33 | 37 | 2380 | 6.01 | Female |
| 13 | FGR | United States | gHTN, gestational thrombocytopenia | 49 | 37 | 2135 | 2.59 | Female |
| 14 | FGR | United States | Crohn’s disease, opioid use, PreE | 30 | 38 | 2425 | 3.98 | Male |
| 15 | FGR | United States | gDM, PreE, anemia | 29 | 36 | 1430 | 0.14 | Male |
| 16 | FGR | United States | gHTN, subclinical hypothyroidism, generalized anxiety, depression | 36 | 37 | 1960 | 0.98 | Male |
| 17 | PM-FGR | Uganda | anemia | 18 | 42 | 2900 | 6.25 | Female |
| 18 | PM-FGR | Uganda | none | 16 | 39 | 2540 | 3.14 | Female |
| 19 | PM-FGR | Uganda | none | 20 | 39 | 2500 | 4.15 | Female |
| 20 | PM-FGR | Uganda | none | 20 | 37 | 2030 | 1.85 | Female |
| 21 | PM-FGR | Uganda | anemia | 17 | 41 | 3000 | 7.00 | Male |
| 22 | PM-FGR | Uganda | anemia | 16 | 39 | 2550 | 3.71 | Male |
| 23 | PM-FGR | Uganda | none | 16 | 40 | 2670 | 3.27 | Male |
| 24 | PM-FGR | Uganda | anemia | 18 | 39 | 2650 | 4.01 | Male |
